# Review of Sterilization Techniques for Medical and Personal Protective Equipment Contaminated With SARS-CoV-2

**DOI:** 10.1109/ACCESS.2020.3002886

**Published:** 2020-06-16

**Authors:** Abbas Johar Jinia, Noora Ba Sunbul, Christopher A. Meert, Cameron A. Miller, Shaun D. Clarke, Kimberlee J. Kearfott, Martha M. Matuszak, Sara A. Pozzi

**Affiliations:** 1 Department of Nuclear Engineering and Radiological SciencesUniversity of Michigan 1259 Ann ArborMI48109-2104USA; 2 Department of Radiation OncologyUniversity of Michigan Medical School 12266 Ann ArborMI48109-5010USA

**Keywords:** COVID-19, medical equipment, PPE, SARS-CoV-2, sterilization

## Abstract

The outbreak of the novel coronavirus disease, COVID-19 turned into a global pandemic in March 2020. During these unprecedented times, there is an increased demand in medical and personal protective equipment (PPE). Since the supplies may take a long time to meet the global demand, reusing PPEs will help health care workers in their response to the COVID-19 pandemic. To ensure the safety and well-being of the medical first responders, PPE needs to be sterilized before reuse. In this review, we examine various sterilization techniques that can be used to sterilize PPEs and point out its limitations. The objective is to provide a foundation of knowledge incorporating different sterilization techniques that allow hospitals and clinics to pick the most suitable technique for sterilization of a particular PPE.

## I. Introduction

The 2019 outbreak of the novel coronavirus disease, COVID-19, in the city of Wuhan, China, turned into a global pandemic in March 2020. During these unprecedented times, health care workers are facing an imminent shortage of personal protective equipment (PPE) and other essential medical supplies [1]. The World Health Organization (WHO) director Dr. Ghebreyesus said, “*We can’t stop COVID-19 without protecting health workers first*” [1]. Since supplies may take a long time to meet the increased global demand, it is prudent that we explore sterilization approaches that would permit recycling and reuse of PPEs for the medical first responders, to help in their response to the COVID-19 pandemic. Successful approaches will result in a complete biological decontamination without any quality deterioration of the PPE. There are several processes available for the sterilization of medical products, including chemicals, heat, ultraviolet radiation, and ionizing radiation.

In this COVID-19 pandemic, PPEs such as N-95 face-piece respirators (FFR), isolation gowns, eye goggles and face shields are used to ensure the safety of the health care workers. There are disposable and reusable isolation gowns commercially available. Disposable gowns are meant for one time use while reusable gowns can be used on multiple patients after following an appropriate process. This process includes laundering, drying and/or sterilization. Details on laundering and drying medical textiles can be found in [2]. Dry steam (121°C for 30 minutes) is commonly used for sterilizing medical textiles [2]. The U.S. Centers for Disease Control and Prevention (CDC) recommends wiping down of face shields and eye goggles with a disinfectant wipe or clean cloth saturated with EPA-registered hospital disinfectant solution prior reuse [3]. N-95 FFRs are typically labelled as “single-use”. However, in these undesired times, reusing FFR will help meet the global demand.

The purpose of this review is to examine existing sterilization techniques through survey of existing literature, recommendations from the IAEA, the U.S. CDC and the U.S. Food and Drug Administration (FDA). Based on our survey findings we compare the limitations of different sterilization techniques, and identify applications where ionizing radiation may hold the most promise. The structure of this review is as follows. Since there exist limited literature on the sterilization of SARS-CoV-2, an understanding of the novel coronavirus SARS-CoV-2 can provide context for finding surrogates to SARS-CoV-2, and thus our review begins with this topic. The following section provides an overview of various sterilization techniques with a particular emphasis on those that have demonstrated capability to inactivate viral population below detectability. We conclude with a discussion of the limitations and challenges, and thoughts on the implementation of ionizing radiation for sterilization of PPEs.

## II. Characteristics and Surrogates of SARS-CoV-2

The COVID-19 disease is caused by a novel coronavirus (CoV), which is a member of the family of viruses that cause severe acute respiratory syndrome (SARS). The WHO refers to the virus as SARS-CoV-2. CoVs are enveloped, positive, single-stranded RNA (ssRNA) viruses with a crown-like appearance under an electron microscope [4]. SARS-CoV-2 has a round or elliptic structure with a diameter of approximately 60-140 nm and its genome structure is approximately 30 kb in length [4]. While there is limited literature available on decontamination of SARS-CoV-2, it is possible to identify appropriate surrogates that have been investigated in the past. To date, seven human CoVs (HCoVs) have been identified, including SARS-CoV-2 [5]. All seven of them cause respiratory diseases in humans. Four of the HCoVs, HCoV-229E, HCoV-OC43, HCoV-NL63 and HKU1, cause mild upper respiratory disease, and in rare cases can cause severe infection in infants, young children and elders. SARS-CoV, identified in 2003 and MERS-CoV, identified in 2012 cause severe respiratory problems and mainly affect the lower respiratory tract [5].

A recent study evaluated the stability and decay rates of SARS-CoV-2 in aerosols and on various surfaces [6]. It was concluded that SARS-CoV-2 was more stable on plastic and stainless steel than on copper and cardboard. The viral activity in the titer that was applied to the plastic surface, was significantly reduced from 10^3.7^ to 10^0.6^ TCID_50_ viruses per mL of medium after 72 hours, implying that the virus was still active and detectable up to 72 hours. On a stainless-steel surface, the same log reduction was observed after 48 hours. For sterilization of medical and personal protective equipment, an established sterility assurance level (SAL) must be satisfied. The log reduction to reach the SAL may be as little as 5 log but depending on the initial viral activity, as much as 12 log reduction may be required [7]. Higher log reduction is always desirable and advantageous. The U.S. FDA requires a 6 log reduction to reach SAL for medical devices labelled sterile. In case of devices intended only for contact with intact skin, FDA recommends a 3 log reduction to attain SAL [8]. With a lifespan that could be more than 72 hours on plastic surfaces, it is essential that we explore techniques that could permit timely reuse of PPE as part of the response to the COVID-19 pandemic.

## III. Sterilization Using Chemicals

Chemicals are mainly used as disinfectants for equipment at hospitals. There are numerous chemicals that are used as disinfectants and it is beyond the scope of this manuscript to review all chemical disinfectants. Some chemicals such as hydrogen peroxide, formaldehyde and glutaraldehyde have also been used as sterilants to inactivate viruses including SARS-CoV [9]–[14]. Hydrogen peroxide produces hydroxyl radicals that are responsible for the damage of DNA and other essential cell components [15]. Formaldehyde works by alkylating the amino and sulfhydral groups of proteins and ring nitrogen atoms of purine bases whereas glutaraldehyde works by alkylating sulfhydral, hydroxyl, carboxyl, and amino groups [15].

### A. Hydrogen Peroxide

Hydrogen peroxide (both vaporized and gas plasma) is an established method that is used to sterilize medical equipment. There exist a wealth of research relating to inactivation of viruses and bacteria spores using hydrogen peroxide [10], [16]–[19].

In a report submitted to the U.S. FDA by a Columbus based company, Battelle, it was stated that vaporized hydrogen peroxide (HPV) can be used to safely sterilize N-95 FFR [20]. In their study, the company used Geobacillus stearothermophilus bacteria spores, as a biological indicator, to contaminate N-95 FFRs (contaminated using either liquid droplets or aerosol exposure). Following the HPV exposure, a 6 log reduction was observed in the biological indicator while maintaining the integrity and performance of the FFR. In this COVID-19 pandemic, Battelle has received an Emergency Use Authorization (EUA) from the FDA to use their HPV based decontamination system for the sterilization of N-95 masks [21].

### B. Limitations

It is not feasible to generalize the limitations and attributes of all chemicals. Some chemicals may be toxic and may also leave stain or odor on the equipment post sterilization, while other chemicals may not. Therefore, discussing the limitations of individual chemicals will provide broader context on selecting the most appropriate chemical sterilant.

Glutaraldehyde is a relatively inexpensive chemical and has an excellent material compatibility [22]. However, the vapors from glutaraldehyde may cause severe respiratory irritation to the operator and therefore, constant monitoring of the glutaraldehyde vapor is recommended. The chemical has a relatively slow mycobactericidal activity and may leave a pungent and irritating odor post sterilization. Glutaraldehyde when touched may result in allergic dermatitis [22].

While hydrogen peroxide has several attributes including environmental friendly, leaves no toxic residuals, and, can be used for temperature and moisture sensitive equipment, there are limitations to this technique. Hydrogen peroxide gas plasma and vaporized hydrogen peroxide may require qualified personnel and state-of-the-art decontamination systems. The chemical is not suitable for certain materials such as, cellulose (paper) and linens. High levels of hydrogen peroxide (greater than 1 ppmTWA) may be toxic and therefore, additional care is required [15].

## IV. Heat

A very recent study measured the stability of SARS-CoV-2 virus at different temperatures [23]. A total of 10^6.8^ TCID_50_ viruses per mL of viral titer was used in this study. It was observed that the SARS-CoV-2 virus is highly stable at 4°C. At this low temperature, the viral concentration was reduced to only 10^6.1^ TCID_50_ per mL on day 14. At 70°C, the SARS-CoV-2 virus was inactivated in 5 minutes. This study provides context to explore the effectiveness of heat on sterilization of PPEs.

### A. Previous Work

A stability study was performed in the past to observe the survival ability of SARS-CoV-P9 virus (10^6^ TCID_50_ in 100 $\mu \text{L}$) at different temperatures [24]. The authors observed that the cytopathogenic effect (CPE) i.e. structural changes in host cell resulting from viral infection became weaker and undetectable after incubation at 56°C for 90 minutes. The study also showed that higher temperatures, 67°C and 75°C, could significantly reduce the viral activity in about 15 minutes. 26-50% cells with CPE were detected after incubation at 67°C and less than 25% cells with CPE were detected after incubation at 75°C.

Darnell *et al.* also tested the ability of heat treatment to inactivate SARS-CoV virus ($320~\mu \text{L}$ aliquots of virus) [13]. During the test, the virus was incubated in 1.5 mL polypropylene cryotubes at three different temperatures (56, 65 and 75°C). Darnell *et al.* study showed that at 56°C, a 5 log reduction in the viral population can be achieved after 20 minutes of incubation. The same log reduction was achieved in nearly 5 minutes when the virus was incubated at 65°C.

In 2004, Rabenau *et al.* studied the stability of different HCoVs including, SARS-CoV, at different temperatures [14]. Rabenau *et al.* study demonstrated that at 56°C, the SARS-CoV viral activity is reduced by a 5 log reduction factor in 30 minutes. The work done by researchers in the past demonstrates the effectiveness of 56°C in inactivating SARS-CoV.

A study at Delft University of Technology in Netherlands, demonstrated that steam sterilization can be used as a simple, cost-effective sterilization technique [25]. In this study the investigators sterilized FFP2 masks using dry sterilization process as well as with regular steam. The masks were exposed to steam at 121°C for 15 minutes. The experiments conducted at the Delft University indicated that the filtration capability of the mask is not compromised by the steam treatment while deactivating the virus.

### B. Limitations

The dependence of virus stability on temperature has permitted the use of heat for sterilization applications. While heat sterilization has many positive attributes, including non-toxicity and easy control and monitor, there are limitations to this technique. Certain medical equipment are heat sensitive, therefore heating may deteriorate the equipment’s performance. Steam sterilization may leave instruments wet, causing them to rust. Repeated heating may damage the equipment permanently, precluding its reuse [15].

## V. Ultraviolet (UV) Radiation

UV radiation can significantly affect the normal state of life by inducing single as well as double DNA strand breaks. When significant double DNA strand breaks occur, it can lead to the loss of genetic material [26]. The effectiveness of UV radiation in sterilization applications is dependent on the wavelength of UV light. The maximum absorption for DNA and RNA occurs at a wavelength of 260 nm and therefore, exposure to UV light around 260 nm wavelength is desired [27].

### A. Previous Work

Duan *et al.* tested the effect of UV radiation on the deactivation of SARS coronavirus [24]. A total of 10^6^ TCID_50_ viruses in $100~\mu \text{L}$ of viral titer were irradiated at 260 nm wavelength UV light. It was observed that the number of cells with CPE was reduced from 50-75% to less than 25% in only 15 minutes of UV irradiation. The CPE was undetectable after 60 minutes of UV exposure. In a related study by Darnell el al., the SARS-CoV virus (10^6.33^ TCID_50_ per mL) was deactivated after 10 minutes of 200-280 nm UV irradiation [13]. Duan *et al.* and Darnell *et al.* used viral titer to study the effectiveness of UV irradiation on virus inactivation; however, when sterilizing PPEs the virus will be present on a surface.

Surfaces may become contaminated with viruses either through infectious body fluids or the settling of airborne viral particles. The contaminated surfaces, when touched by healthy people, may result in the transmission of the virus. Therefore, it is crucial that we explore the usefulness of a sterilization technique in killing the virus on surfaces. In a study performed by Tseng and Li, different viruses, including ssRNA, were inactivated on the surface of gelatin-based medium using a UV source with a radiation peak at 253.7 nm [28]. The study concluded that the survival fractions of viruses is inversely proportional to UV dose. 90% reduction in the ssRNA viral population can be achieved using low UV dose of 1.32 to 3.20 mJ/cm^2^. Although there is limited literature available on the inactivation of SARS-CoV virus using UV light, researchers have investigated deactivation of other viruses, such as H1N1 and H5N1 (both RNA viruses), on N-95 FFR [29]–[31].

### B. Limitations

The use of UVC (200-280 nm) radiation has been successful in inactivating various viruses. UV radiation can inactivate the virus on the surface that is directly exposed to UV light. Shorter wavelength UV radiation, such as UVC radiation, is not highly penetrating [32] and therefore, it may be ineffective in sterilizing the inner layers of certain PPEs, such as N-95 FFR pores. Human exposure to UVC can cause severe skin diseases and therefore, additional care is necessary to avoid human exposure to UV light (UVC is the most damaging type of UV radiation) [32].

## VI. Gamma/X-Ray Irradiation

Ionizing radiation, such as high energy X-rays/gamma radiation will damage DNA either by direct energy deposition or by secondary interactions with the surrounding atoms or molecules. In particular, secondary interactions occur with surrounding water molecules, leading to the formation of OH^−^ free radicals that are responsible for 90% of the resulting DNA damage [33]. Both direct and indirect interactions can cause significant double strand breaks often resulting in cell death.

### A. Previous Work

Like other sterilization methods, gamma irradiation is governed by an ISO standard: ISO 11137 [34]. Although SARS-CoV-2 was not studied in the past, there is a wealth of research relating to virus inactivation using gamma irradiation. Most recently, a publication by Feldmann *et al.* [35] irradiated several viruses, including tissues infected with SARS-CoV to gamma rays produced by a commercial cobalt-60 (Co-60) source. The study subjected the SARS-CoV cells to doses between 10 kGy and 40 kGy, and tested samples in triplicate to observe inactivation of viral activity. Even at the lowest dose of 10 kGy, the SARS-CoV was completely inactivated - meaning that no virus growth was observed in post-irradiation tissue assays. This value is much lower than the ISO 11137 standard of 25 kGy. The authors specifically note the large genomic complexity of the SARS-CoV virus and mention that inactivation is inversely correlated to genome size. The authors recommend a 20 kGy dose, accounting for a 2x safety factor to inactivate the SARS-CoV virus.

A related work by Darnell *et al.* [13] also subjected SARS-CoV to gamma irradiation produced by a Co-60 source; however, the maximum dose was only 150 Gy. The author comments that there was no change in virus infectivity after irradiation of 150 Gy. The work by Darnell *et al.* may not determine the dose required to reach a SAL, but it can establish a starting point to determine the D_10_, which can then be used to estimate dose required to reach a desired SAL.

A publication by Jebri *et al.* [36] discusses that gamma ray sensitivity is dependent on the infected medium: independent samples of MS2 bacteriophages in water, autoclaved raw sewage, and a tryptone solution were exposed to gamma rays from a Co-60 source and observed that the dose required for a 1 log reduction, the D_10_, was 0.5 kGy, but 1.0 kGy in autoclaved raw sewage, and 1.2 kGy in 1% tryptone. Because the infected PPE will be relatively dry, it is possible that a higher dose will be required than those discussed in the literature.

When using ionizing radiation, we must consider the effect of the radiation on the PPE itself. It is known that ionizing radiation can damage polymeric materials by causing cross-linking or scissioning the molecules [33]. These chemical changes may result in embrittlement, loss of tensile strength, and loss of molecular weight, all depending on the polymer being irradiated. In the case of N-95 masks, the most critical component is the filter itself, designed to filter 95% of particles of size $0.3~\mu \text{m}$. The filters rely upon electrostatic processes to filter particles. Recent works by Cramer *et al.* [37] and Man el al. [25] showed that with doses as low as 10 kGy, the filter efficiency is significantly reduced. The work by Cramer *et al.* and Man *et al.* provided context to explore the effectiveness of low radiation doses (less than 10 kGy) on sterilization applications.

### B. Application of a Varian M9 Electron Linear Accelerator

Electron linear accelerators, referred to as linacs, are used at hospitals and clinics around the world for radiation therapies. When the linac is not being used for treating patients, hospitals may consider using its linac for sterilization applications. We performed Monte Carlo simulations on a commercially available linac to provide context on the potential use of a linac for PPE sterilization at the hospitals.

**FIGURE 1.**
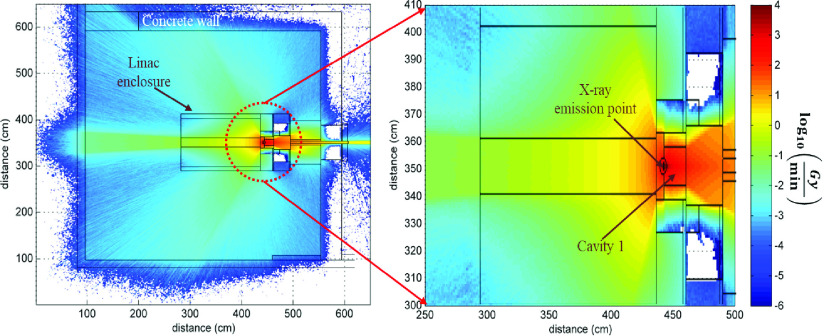
Top down cross section of the shielded Varian M9 electron linear accelerator at UM.

At the University of Michigan (UM), we have a Varian M9 9 MeV linac [38] that is currently licensed to operate at a frequency of 50 Hz, producing an average electron current of $20~\mu \text{A}$. Under these licensed operating conditions, the X-rays produced can deliver dose rates sufficient for PPE sterilization in minutes to hours. A cylindrical cavity (14 cm diameter, 20 cm length), available next to the X-ray emitter, can hold numerous PPEs (exact number depends on the size of the PPE and its packaging) during a single irradiation cycle (**FIGURE 1**). MCNPX [39] simulations show that a dose greater than 5 kGy can be delivered in the cylindrical cavity in approximately 30 minutes. If the linac is operated at the maximum repetition rate of 250 Hz, estimated irradiation times would decrease by a factor of 5.

**FIGURE 2.**
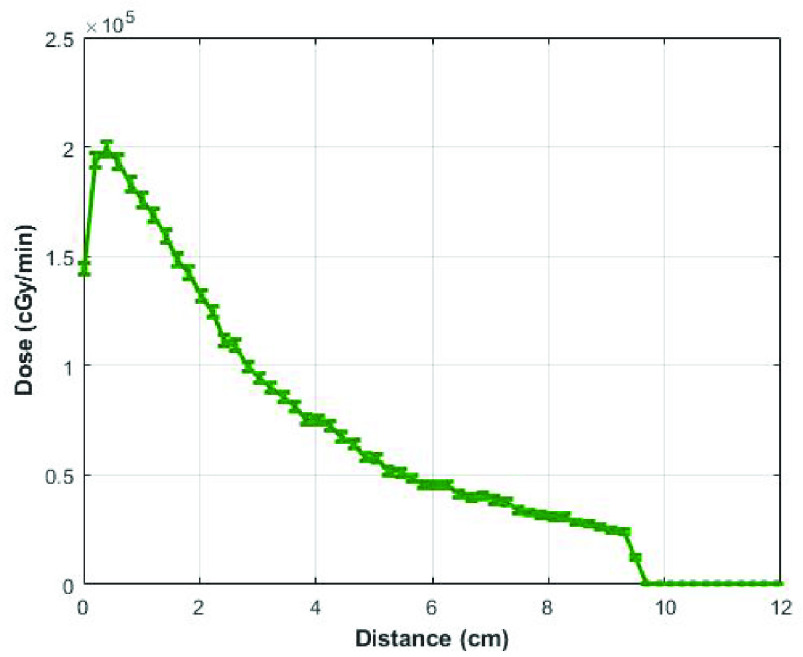
Percent depth dose (PDD) curve in the cubical container, scored along the center $2\times 2\,\,\text {mm}^{2}$ beam axis.

**FIGURE 3.**
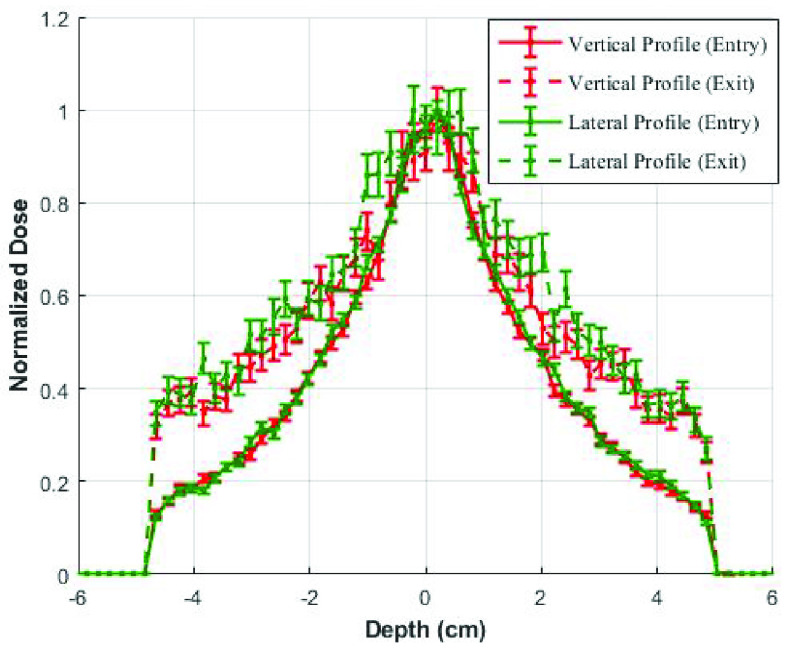
Lateral and vertical dose distributions at the entry and exit of the cubical container.

A homogeneous dose distribution is required to ensure that the PPEs are evenly irradiated. Another MCNPX simulation was performed to evaluate the dose distribution in the cylindrical cavity. Results show that the dose distribution, in a small 9.5 cm cubical container filled with reference material water, exhibits heterogeneity along the beam (through percent depth dose evaluation) and across the beam directions (through beam profile evaluations). The percent depth dose (PDD) curve (**FIGURE 2**) exhibited decrease in dose as the photon radiation penetrated deeper into the cubical container. Therefore, it is recommended to irradiate the PPE from both sides to ensure maximum and uniform dose delivery. In case of flattening filter free (FFF) accelerator, there exist inhomogeneity in the beam profile (**FIGURE 3**). The inhomogeneity can be compensated through a beam filter that will improve the homogeneity of the dose distribution across the beam direction.

### C. Limitations

Sterilization processes using gamma/X-ray irradiation have several advantages, including uniform dose delivery without opening product packaging and lack of residue or induced radioactivity post-irradiation. Because ionizing radiation, especially high-energy X-rays and gamma rays, are highly penetrating, they can travel through the outer surface of PPEs, resulting in effective inactivation of viral cells. Radiation damage to the constituent materials of the PPE must be considered when using ionizing radiation for sterilization applications. IAEA recommends using ionizing radiation for sterilization of surgical mask and gloves [40].

Regulatory restrictions (for safety purposes) exist on the amount of dose that can be delivered to the public and radiation workers. To meet the regulatory requirement, appropriate shielding of the radiation source is required. The shielding design must allow for high dose delivery to the PPE while reducing the dose received by the radiation worker.

## VII. Conclusion

During a pandemic, hospitals face shortage of PPEs and other essential medical equipment. Since the supply is unable to meet the demand in these unprecedented times, reusing PPE is a potentially feasible option. To ensure the safety and well-being of the medical personnel, PPE needs to be sterilized before reuse. Sterilization processes should not compromise the quality and performance of the PPE itself.

The basic principle and limitations of existing sterilization techniques that are used for the sterilization of medical and personal protective equipment have been reviewed for effectiveness, performance degradation of the PPE, and typical treatment times (doses in case of ionizing radiation). Our objective was to provide a foundation of knowledge incorporating different sterilization techniques that allows one to pick the most suitable technique for sterilization of a particular PPE during a global pandemic.

Chemicals are widely used as disinfectants at hospitals but there is limited literature available on chemicals being used as sterilants. While heat may seem the most convenient and cost-effective technique, this sterilization process is not suitable for temperature and moisture sensitive equipment. The inability of UV radiation to penetrate deep into the inner layers makes this technique less reliable for certain PPEs. Ionizing radiation help overcome some of the above mentioned limitations and therefore, further investigation is needed to explore the effectiveness of this technique.

The findings from this review can provide hospitals with a technique that could be used to sterilize PPEs. Lower radiation doses (less than 10 kGy) allow hospitals to operate safely without worrying about high dose delivery to the staff and patients. It also permits hospitals to operate under their current licensing agreement reducing regulatory burden. With a sterilization technique available in house, hospitals can save time and continue to serve patients with high quality sterilized PPEs.

## References

[1] WHO. Shortage of Personal Protective Equipment Endangering Health Workers Worldwide. Accessed: Apr. 15, 2020. [Online]. Available: https://www.who.int/news-room/detail/03-03-2020-shortage-of-personal-protective-equipment-endangering-health-workers-worldwide

[2] H. M. Zins and M. Howard, “Reusable medical textiles,” in Handbook of Medical Textiles. Sawston, U.K.: Woodhead, 2011, pp. 80–105.

[3] U.S. Centers for Disease Control and Prevention. (2020). Strategies for Optimizing the Supply of Eye Protection. CDC Website. Accessed: 5 28, 2020. [Online]. Available: https://www.cdc.gov/coronavirus/2019-ncov/hcp/ppe-strategy/eye-protection.html

[4] M. Cascella, M. Rajnik, A. Cuomo, S. C. Dulebohn, and R. Di Napoli, “Features, evaluation and treatment coronavirus (COVID-19),” in StatPearls, Treasure Island, FL, USA: StatPearls Publishing, 2020. 32150360

[5] Y. Chen, Q. Liu, and D. Guo, “Emerging coronaviruses: Genome structure, replication, and pathogenesis,” J. Med. Virology, vol. 92, no. , pp. 418–423, Apr. 2020, doi: 10.1002/jmv.25681. 31967327 PMC7167049

[6] N. van Doremalen, T. Bushmaker, D. H. Morris, M. G. Holbrook, A. Gamble, B. N. Williamson, A. Tamin, J. L. Harcourt, N. J. Thornburg, S. I. Gerber, J. O. Lloyd-Smith, E. de Wit, and V. J. Munster, “Aerosol and surface stability of SARS-CoV-2 as compared with SARS-CoV-1,” New England J. Med., vol. 382, no. 16, pp. 1564–1567, Apr. 2020, doi: 10.1056/NEJMc2004973. 32182409 PMC7121658

[7] A. Hume, J. Ames, L. Rennick, W. Duprex, A. Marzi, J. Tonkiss, and E. Mühlberger, “Inactivation of RNA viruses by gamma irradiation: A study on mitigating factors,” Viruses, vol. 8, no. 7, p. 204, Jul. 2016, doi: 10.3390/v8070204. PMC4974539 27455307

[8] U. S. Food and D. Administration, “Submission and review of sterility information in premarket notification (510(k)) submissions for devices labeled as sterile guidance for industry and food and drug administration staff,” U.S. Food, Drug Admin., Silver Spring, MD, USA, Tech. Rep., 2016. [Online]. Available: https://www.fda.gov/regulatory-information/search-fda-guidance-documents/submission-and-review-sterility-information-premarket-notification-510k-submissions-devices-labeled

[9] S. N. Rudnick, J. J. McDevitt, M. W. First, and J. D. Spengler, “Inactivating influenza viruses on surfaces using hydrogen peroxide or triethylene glycol at low vapor concentrations,” Amer. J. Infection Control, vol. 37, no. 10, pp. 813–819, Dec. 2009, doi: 10.1016/j.ajic.2009.06.007. PMC7115294 19822378

[10] R. A. Heckert, M. Best, L. T. Jordan, G. C. Dulac, D. L. Eddington, and W. G. Sterritt, “Efficacy of vaporized hydrogen peroxide against exotic animal viruses.,” Appl. Environ. Microbiol., vol. 63, no. 10, pp. 3916–3918, 1997, doi: 10.1128/aem.63.10.3916-3918.1997. 9327555 PMC168702

[11] L. Hall, J. A. Otter, J. Chewins, and N. L. Wengenack, “Use of hydrogen peroxide vapor for deactivation of mycobacterium tuberculosis in a biological safety cabinet and a room,” J. Clin. Microbiol., vol. 45, no. 3, pp. 810–815, Mar. 2007, doi: 10.1128/JCM.01797-06. 17166957 PMC1829131

[12] D. J. Viscusi, M. S. Bergman, B. C. Eimer, and R. E. Shaffer, “Evaluation of five decontamination methods for filtering facepiece respirators,” Ann. Occup. Hyg., vol. 53, no. 8, pp. 815–827, 2009, doi: 10.1093/annhyg/mep070. 19805391 PMC2781738

[13] M. E. R. Darnell, K. Subbarao, S. M. Feinstone, and D. R. Taylor, “Inactivation of the coronavirus that induces severe acute respiratory syndrome, SARS-CoV,” J. Virolog. Methods, vol. 121, no. 1, pp. 85–91, Oct. 2004, doi: 10.1016/j.jviromet.2004.06.006. PMC7112912 15350737

[14] H. F. Rabenau, J. Cinatl, B. Morgenstern, G. Bauer, W. Preiser, and H. W. Doerr, “Stability and inactivation of SARS coronavirus,” Med. Microbiol. Immunology, vol. 194, nos. 1–2, pp. 1–6, Jan. 2005, doi: 10.1007/s00430-004-0219-0. 15118911 PMC7086689

[15] U.S. Centers for Disease Control and Prevention. (2008). Guideline for Disinfection and Sterilization in Healthcare Facilities. Miscellaneous Inactivating Agents. Accessed: Apr. 16, 2020. [Online]. Available: https://www.cdc.gov/infectioncontrol/guidelines/disinfection/

[16] W. Bar, G. Marquezdebar, A. Naumann, and S. Ruschgerdes, “Contamination of bronchoscopes with mycobacterium tuberculosis and successful sterilization by low-temperature hydrogen peroxide plasma sterilization,” Amer. J. Infection Control, vol. 29, no. 5, pp. 306–311, Oct. 2001, doi: 10.1067/mic.2001.117040. 11584256

[17] M. S. Kyi, J. Holton, and G. L. Ridgway, “Assessment of the efficacy of a low temperature hydrogen peroxide gas plasma sterilization system,” J. Hosp. Infect., vol. 31, no. 4, pp. 275–284, 1995, doi: 10.1016/0195-6701(95)90206-6. 8926377

[18] K. Vickery, A. K. Deva, J. Zou, P. Kumaradeva, and L. B. A. Y. E. Cossart, “Inactivation of duck hepatitis b virus by a hydrogen peroxide gas plasma sterilization system: Laboratory and ‘in use’ testing,” J. Hospital Infection, vol. 41, no. 4, pp. 317–322, Apr. 1999, doi: 10.1053/jhin.1998.0516. 10392338

[19] M. Grare, M. Dailloux, L. Simon, P. Dimajo, and C. Laurain, “Efficacy of dry mist of hydrogen peroxide (DMHP) against mycobacterium tuberculosis and use of DMHP for routine decontamination of biosafety level 3 laboratories,” J. Clin. Microbiol., vol. 46, no. 9, pp. 2955–2958, Sep. 2008, doi: 10.1128/JCM.00250-08. 18632916 PMC2546749

[20] Battelle. (2016). Final Report for the Bioquell Hydrogen Peroxide (HPV) Decontamination for Resue of N95 Respirators. [Online]. Available: https://www.fda.gov/media/136386/download

[21] Battelle. FDA Letter of Approval for Emergency Use Authorization for the Battelle Decontamination System, an HPV System. Accessed: Apr. 15, 2020. [Online]. Available: https://www.battelle.org/docs/default-source/commercial-offerings/industry-solutions/battelle-eua.pdf

[22] W. A. Rutala and D. J. Weber, “Disinfection and sterilization: An overview,” Amer. J. Infection Control, vol. 41, no. 5, pp. S2–S5, 5 2013, doi: 10.1016/j.ajic.2012.11.005. 23622742

[23] A. Chin, J. Chu, M. Perera, K. Hui, H.-L. Yen, M. Chan, M. Peiris, and L. Poon, “Stability of SARS-CoV-2 in different environmental conditions,” Lancet Microbe, vol. 1, no. 1, p. e10, 5 2020. [Online]. Available: https://www.sciencedirect.com/science/article/pii/S2666524720300033?via%3Dihub doi: 10.1016/s2666-5247(20)30003-3. 32835322 PMC7214863

[24] S.-M. Duan, X. S. Zhao, R. F. Wen, J.-J. Huang, G. H. Pi, S. X. Zhang, J. Han, S. L. Bi, L. Ruan, and X.-P. Dong, “Stability of SARS coronavirus in human specimens and environment and its sensitivty to heating and UV irradiation,” Biomed. Environ. Sci., vol. 16, pp. 246–255, Sep. 2003. 14631830

[25] P. de Man, B. van Straten, J. van den Dobbelsteen, A. van der Eijk, T. Horeman, and H. Koeleman, “Sterilization of disposable face masks by means of standardized dry and steam sterilization processes; An alternative in the fight against mask shortages due to COVID-19,” J. Hospital Infection, vol. 105, no. 2, pp. 356–357, Jun. 2020, doi: 10.1016/j.jhin.2020.04.001. PMC7194556 32277964

[26] R. P. Rastogi, Richa, A. Kumar, M. B. Tyagi, and R. P. Sinha, “Molecular mechanisms of ultraviolet radiation-induced DNA damage and repair,” J. Nucleic Acids, vol. 2010, pp. 1–32, Dec. 2010, doi: 10.4061/2010/592980. PMC3010660 21209706

[27] J. G. Anderson, N. J. Rowan, S. J. MacGregor, R. A. Fouracre, and O. Farish, “Inactivation of food-borne enteropathogenic bacteria and spoilage fungi using pulsed-light,” IEEE Trans. Plasma Sci., vol. 28, no. 1, pp. 83–88, Feb. 2000, doi: 10.1109/27.842870.

[28] C.-C. Tseng and C.-S. Li, “Inactivation of viruses on surfaces by ultraviolet germicidal irradiation,” J. Occupational Environ. Hygiene, vol. 4, no. 6, pp. 400–405, Apr. 2007, doi: 10.1080/15459620701329012. PMC7196698 17474029

[29] D. Mills, D. A. Harnish, C. Lawrence, M. Sandoval-Powers, and B. K. Heimbuch, “Ultraviolet germicidal irradiation of influenza-contaminated N95 filtering facepiece respirators,” Amer. J. Infection Control, vol. 46, no. 7, pp. e49–e55, Jul. 2018, doi: 10.1016/j.ajic.2018.02.018. PMC7115285 29678452

[30] M. B. Lore, B. K. Heimbuch, T. L. Brown, J. D. Wander, and S. H. Hinrichs, “Effectiveness of three decontamination treatments against influenza virus applied to filtering facepiece respirators,” Ann. Occup. Hyg., vol. 56, no. 1, pp. 92–101, 2012, doi: 10.1093/annhyg/mer054. 21859950

[31] B. K. Heimbuch, W. H. Wallace, K. Kinney, A. E. Lumley, C.-Y. Wu, M.-H. Woo, and J. D. Wander, “A pandemic influenza preparedness study: Use of energetic methods to decontaminate filtering facepiece respirators contaminated with H1N1 aerosols and droplets,” Amer. J. Infection Control, vol. 39, no. 1, pp. 1–9, 2011, doi: 10.1016/j.ajic.2010.07.004. 21145624

[32] WHO. Ultraviolet Radiation and the INTERSUN Programme. Accessed: Apr. 15, 2020. [Online]. Available: https://www.who.int/uv/faq/whatisuv/en/index2.html

[33] IAEA. (2008). Trends in Radiation Sterilization of Health Care Products. [Online]. Available: https://www.iaea.org/publications/7691/trends-in-radiation-sterilization-of-health-care-products

[34] Sterilization of Health Care Products—Radiation—Part 1: Requirements for Development, Validation and Routine Control of a Sterilization Process for Medical Devices. Accessed: Apr. 15, 2020. [Online]. Available: https://committee.iso.org/standard/33952.html

[35] F. Feldmann, W. L. Shupert, E. Haddock, B. Twardoski, and H. Feldmann, “Gamma irradiation as an effective method for inactivation of emerging viral pathogens,” Amer. J. Tropical Med. Hygiene, vol. 100, no. 5, pp. 1275–1277, 5 2019, doi: 10.4269/ajtmh.18-0937. PMC6493948 30860018

[36] S. Jebri, F. Hmaied, J. Jofre, MariemYahya, J. Mendez, I. Barkallah, and M. Hamdi, “Effect of gamma irradiation on bacteriophages used as viral indicators,” Water Res., vol. 47, no. 11, pp. 3673–3678, Jul. 2013, doi: 10.1016/j.watres.2013.04.036. 23726703

[37] A. Cramer, E. Tian, H. Y. Sherryl, M. Galanek, E. Lamere, J. Li, R. Gupta, and M. P. Shor, “Disposable N95 masks pass qualitative fit-test but have decreased filtration efficiency after cobalt-60 gamma irradiation,” MedRxiv, to be published. [Online]. Available: https://www.medrxiv.org/content/10.1101/2020.03.28.20043471v1.full.pdf+html

[38] VAREX Imaging. Linatron M9 & M9A Modular High-Energy X-Ray Source. Accessed: Apr. 15, 2020. [Online]. Available: https://www.vareximaging.com/products/security-industrial/linear-accelerators/linatron-m/linatron-m9

[39] D. B. Pelowitz, Mcnp6 User’S Manual. Santa Fe, NM, USA: Los Alamos National Laboratory, 2013.

[40] IAEA. Radiation Effective in Sterilizing Personal Protective Equipment Except For Respiratory Masks—IAEA. Accessed: 5 3, 2020. [Online]. Available: https://www.iaea.org/newscenter/pressreleases/radiation-effective-in-sterilizing-personal-protective-equipment-except-for-respiratory-masks-iaea

